# Marked alveolar apoptosis/proliferation imbalance in end-stage emphysema

**DOI:** 10.1186/1465-9921-6-14

**Published:** 2005-02-10

**Authors:** Fiorella Calabrese, Cinzia Giacometti, Bianca Beghe, Federico Rea, Monica Loy, Renzo Zuin, Giuseppe Marulli, Simonetta Baraldo, Marina Saetta, Marialuisa Valente

**Affiliations:** 1 Institute of Pathology, University of Padua, Italy; 2 Department of Clinical and Experimental Medicine, Section of Respiratory Diseases, University of Padua, Italy; 3 Department of Gastroenterological Sciences, Section of Thoracic Surgery, University of Padua, Italy

**Keywords:** apoptosis, proliferation, end-stage emphysema

## Abstract

**Background:**

Apoptosis has recently been proposed to contribute to the pathogenesis of emphysema.

**Methods:**

In order to establish if cell fate plays a role even in end-stage disease we studied 16 lungs (9 smoking-associated and 7 α1antitrypsin (AAT)-deficiency emphysema) from patients who had undergone lung transplantations. Six unused donor lungs served as controls. Apoptosis was evaluated by TUNEL analysis, single-stranded DNA laddering, electron microscopy and cell proliferation by an immunohistochemical method (MIB1). The role of the transforming growth factor (TGF)-β1 pathway was also investigated and correlated with epithelial cell turnover and with the severity of inflammatory cell infiltrate.

**Results:**

The apoptotic index (AI) was significantly higher in emphysematous lungs compared to the control group (p ≤ 0.01), particularly if only lungs with AAT-deficiency emphysema were considered (p ≤ 0.01 vs p = 0.09). The proliferation index was similar in patients and controls (1.9 ± 2.2 vs 1.7 ± 1.1). An increased number of T lymphocytes was observed in AAT-deficiency lungs than smoking-related cases (p ≤ 0.05). TGF-β1 expression in the alveolar wall was higher in patients with smoking-associated emphysema than in cases with AAT-deficiency emphysema (p ≤ 0.05). A positive correlation between TGF-βRII and AI was observed only in the control group (p ≤ 0.005, r^2 ^= 0.8). A negative correlation was found between the TGF-β pathway (particularly TGF-βRII) and T lymphocytes infiltrate in smoking-related cases (p ≤ 0.05, r^2 ^= 0.99)

**Conclusion:**

Our findings suggest that apoptosis of alveolar epithelial cells plays an important role even in end-stage emphysema particularly in AAT-deficiency disease. The TGFβ-1 pathway does not seem to directly influence epithelial turnover in end-stage disease. Inflammatory cytokine different from TGF-β1 may differently orchestrate cell fate in AAT and smoking-related emphysema types.

## Background

Pulmonary emphysema, a significant global health problem, is a pathological condition characterized by enlargement of the airspaces distal to the terminal bronchiole, destruction of the alveolar walls, without and/or with mild fibrosis [1]. To date the pathogenesis remains enigmatic. The most prevailing hypothesis since the 1960s has been the elastase/antielastase imbalance theory of inflammation [2]. Briefly, the concept is that activated inflammatory cells release large quantities of elastases, overwhelming local antiprotease activity with consequent damage to the alveolar wall matrix [3]. However the emphasis on alveolar matrix destruction by a combination of inflammation and excessive proteolysis has failed to fully explain the loss of lung tissue, particularly when compared to alterations seen in other inflammatory lung diseases.

Recently more attention has been paid to alveolar epithelial injury in addition to alveolar matrix destruction. The presence of apoptosis has recently been described in animal models of emphysema [4,5] and in a few studies of human disease [6-9].

The majority of investigations have focused the attention on smoking-related emphysema keeping in mind that cigarette smoking was the main cause of apoptotic cell death. Cigarette smoke may induce alveolar cell apoptosis either directly by a cytotoxic effect on pneumocytes or indirectly by decreasing the production of vascular endothelial growth factor (VEGF) via altered epithelial cells [7]. To date smoking-associated centrilobular emphysema is the only studied form of emphysema in which apoptosis, and more recently also proliferation, have been investigated [9]. Alterations of lung epithelial cell turnover in end-stage emphysema, either smoking-associated emphysema or α1-antitrypsin (AAT)-deficiency emphysema, are up to now not well distinguished.

Moreover apoptotic phenomenon has been previously investigated in moderate/severe smoking-related forms of emphysematous lungs obtained almost exclusively from lung volume reduction surgery [6,7,9]. If cell fate is a stable, progressive and/or a decreasing process in end-stage disease is to date unknown.

Among the growth factors, transforming growth factor (TGF)-β1 could play a crucial role in the remodeling process occurring in emphysematous parenchyma. TGF-β1, other than its known profibrogenetic [10] and anti-inflammatory effects [11,12], has an important influence on epithelial cell growth [14]. It has been demonstrated that it has an inhibitory effect on the growth of lung epithelial cells, particularly for airway epithelium [14,15].

The cytokine has been shown to be over-expressed in patients with a history of smoking and chronic obstructive pulmonary disease (COPD) [16,17]. Paracrine (mainly produced by macrophages) and autocrine (released by epithelial cells) activity of this growth factor could play an important role in the loss of the alveolar walls by inducing apoptotic cell death.

In the present work the degree of apoptotic cell death and epithelial proliferation in the lungs of patients with different types of end-stage emphysema was studied. The severity of inflammatory cell infiltrate (ICI) was also quantified and correlated with epithelial cell turnover. Further, the TGF-β1 pathway was detected and correlated with the apoptotic index (AI), the proliferative index (PI) and the ICI.

## Methods

### Lung tissue preparation

Lung tissue used in the present study comprised material from 16 patients undergoing lung transplantation for end-stage emphysema at the Thoracic Surgery Unit of the University of Padua Medical School. Cold ischemia preservation was 60 minutes and 120 minutes, respectively, for single and double lung transplantations. Small-sized pieces from all lobes were cut and immediately fixed in Karnovsky's solution for electron microscopy. The lungs were then gently fixed in 10% phosphate-buffered formalin by airway perfusion and processed for sectioning (3 μm). Samples were selected from specimens that showed features of excellent tissue preservation and adequate lung inflation. In particular, large thin blocks approximately 30 × 25 mm were cut from the subpleural areas of the apical anterior and lingular segments of the upper lobes, as well as the apical and basal segments of the lower lobes. A more centrally placed block was taken to sample the segmented airways and blood vessels. The right lung was sampled in the same way with the middle lobe being treated in the same way as the lingula [18]. Adult control lungs were obtained from unused donor lungs for transplantation (6 cases). The Local Research Ethics Committee approved the study.

### TUNEL analysis

The terminal deoxynucleotidyl transferase-mediated dUTP-biotin nick end-labeling method (TUNEL) was used to investigate the presence of apoptosis. Sections were processed in accordance with Gavrieli et al's method [19]. Briefly, after deparaffinization and rehydration, sections were digested with proteinase K (Boehringer Mannheim, Mannheim, Germany) at a concentration of 20 μg/ml for 15 minutes. The slides were then incubated with TdT/biotinylated dUTP diluted in buffer (Boehringer Mannheim, Mannheim, Germany). The slides were developed by using diaminobenzidine and 30 ml hydrogen peroxide. For negative controls, some slides were incubated in buffer without TdT or biotinylated UTP. For positive controls, some slides were incubated with 1 μg/ml DNAse (Sigma-Aldrich, Milan, Italy).

### Electron microscopy

Lung specimens fixed in Karnovsky's solution (2% paraformaldehyde, 2.5% glutaraldehyde in Millonig, pH 7.3) for 24 hours were post-fixed with 1% osmium tetroxide (Millonig, pH 6.8) for 1 hour, and then progressively dehydrated in alcohol and embedded in epon. Semi-thin sections were stained with 0.1% toluidine blue for light microscopic examination. Ultra-thin sections were stained with uranyl acetate and lead citrate for transmission electron microscopy performed using a Hitachi H-7000 (Hitachi Ltd., Tokyo, Japan).

### Oligonucleosomal-length DNA laddering

The presence of oligonucleosomal-length DNA cleavage was investigated with APO-DNA1 (Maxim Biotech Inc, San Francisco, CA, USA) in 12 cases (4 AAT-emphysema patients, 4 smoking-related emphysema patients and 4 controls) in which frozen tissue was available. Briefly, DNA was obtained from lung tissue samples using proteinase K-phenol extraction. Dephosphorylated adaptors were ligated to 5' phosphorylated blunt ends with T_4 _DNA ligase to 500 ng of lung sample DNA (for 16 h at 16°C). These then served as primers in LM-PCR under the following conditions: hot start (72°C for 8 min), 30 cycles (94°C for 1 min, and 72°C for 3 min) and extension (72°C for 15 min). Every reaction set included thymus DNA as a positive control and normalization of the amount of reaction products. Amplified DNA was subjected to electrophoresis on 1.2% agarose gel containing ethidium bromide. Images were scanned and the DNA fragmentation levels were based on the density of the bands ranging between 1000 base pairs (bp) and 300 bp. The percentage of DNA fragmentation was quantified by scanning densitometry.

### Immunohistochemistry for TGF-β1, TGF-βRII and MIB1

All lung sections were subjected to antigen retrieval by heating in a microwave oven on high power for 8 minutes in 0.01 mol/l citrate buffer (ph 6.0) and then incubated with a mouse monoclonal anti-TGF-β_1_-β_2 _and-β_3 _primary antibody to active TGF-β1 (150 μg/ml; dilution 1:20, Genzyme Diagnostics, Cambridge, MA), with polyclonal antibody against TGF-β receptor type II (200 μg/ml, dilution 1:200, Santa Cruz Biotechnology Inc., Santa Cruz) and monoclonal MIB-1 antibody (1:50 Dako, Santa Barbara, CA, U.S.A.), which recognizes the Ki-67 antigen in paraffin-embedded tissue sections. Immunohistochemical investigations were done on the sections from the same paraffinembedded specimens processed for TUNEL analysis.

The detection system was the Vectastain ABC kit (Vector Peterborough, UK) with 3-amino-9-ethylcarbazole (for TGF-β1, TGF-βRII) and with a mixture of 3,3'-diamino-benzidine tetra7 hydrochloride (Dako) and hydrogen peroxide as the chromogenic substrates. Sections were counterstained with Mayer's hematoxylin.

### Immunohistochemistry for inflammatory cell infiltrate (ICI)

In all samples, immunohistochemistry for the characterization of ICI was carried out by using the following antibody panel: CD20 (1.100), CD45RO (1.100), CD4 (1:20), CD8 (1:50), CD3 (1:100), CD68 (1:50) (Dako, Santa Barbara, CA, U.S.A.). The detection system was the Vectastain ABC kit, as described above.

For all immunohistochemistry experiments, negative controls were performed by incubation of the sections with the omission of primary antibody and using the antibody diluents alone or the appropriate non-immune IgG in each case.

### Double immune-labeling

For simultaneous detection of DNA fragmentation and cell proliferation a double labeling was also performed. The TUNEL technique was first performed and the staining achieved was diaminobenzidine as chromogen. For MIB1 immunolocalization in the second staining sequence the sections were stained with 5-bromo-4-chloro-3-indoxyl phosphate/nitro blue tetrazolium (BCIP/NBT alkaline phosphatase Kit II, Vector Laboratories (Vector Peterborough, UK).

### Image analysis

Immunoassay for TGF-β1 and TGF-βRII was detected by using digital quantitative analysis (Image Pro Plus software version 4.1, Media Cybernetics, Silver Spring MD) as previously described [13]. Quantification of TUNEL, MIB1 positive cells and ICI was restricted to the alveolar wall. Images for each lung section from the upper and lower lobes were acquired with a 40X lens.

In each case at least 50 microscopic randomly chosen fields were analyzed. A total of 5,000 epithelial cells were counted for AI and PI and the values were expressed as percentages.

### Statistical analysis

To avoid observer bias the cases were coded and measurements were made without knowledge of clinical data. Differences between groups were detected using the analysis of variance for clinical data and the Kruskall-Wallis test for histological data. The Mann-Whitney U test was performed after the Kruskall-Wallis test when appropriate. The statistical tests used were two-sided.

Correlation coefficients were calculated using Spearman's rank method. Probability values of 0.05 or less were accepted as significant. Group data were expressed as means and SD or as medians and range when appropriate.

## Results

### Clinical data and histological findings

Major clinical data for patients with emphysema are shown in Table 1.

**Table 1:** Subject Characteristics

**Case**	**Sex**	**Age**	**Emphysema type**	**Packs/year**	FEV_1*_	FEV_1_/FVC*	**Transplantation**
1	M§	49	AAT deficiency	27	27	27	BSLT *
2	M	59	Smoking	36,5	31	38	RtSLT †
3	F||	62	Smoking	7	17	55	BSLT
4	F	62	Smoking	36,5	13	30	LtSLT ‡
5	M	62	Smoking	108	15	45	BSLT
6	F	49	Smoking	73	12	33	BSLT
7	M	47	Smoking	54	22	60	BSLT
8	M	59	AAT deficiency	36,5	20	56	BSLT
9	M	64	Smoking	54	6	42	LtSLT
10	M	63	Smoking	54	24	37	BSLT
11	M	51	AAT deficiency	54	11	25	BSLT
12	M	53	AAT deficiency	108	8	14	BSLT
13	M	45	AAT deficiency	73	17	24	BSLT
14	M	56	Smoking	36,5	15	32	RtSLT
15	F	41	AAT deficiency	54	35	38	BSLT
16	M	51	AAT deficiency	36,5	34	36	BSLT

*BSLT: bilateral single lung transplantation, † RtSLT: right transplant single lung transplantation, ‡ LtSLT: left transplant single lung transplantation, §M: male, || F: female. FEV_1 _and FVC are given as percentages of predicted values.

Average patient age was 54.4 ± 7.5 years. FEV1 mean was 19 ± 8.9 (predicted for sex, age, and body weight). Bilateral single lung transplantation was performed in 12 out of 16 patients. All patients had been heavy smokers: 7 were only smoking-associated emphysema cases (51 ± 28 packs-year) and 9 were both AAT-deficiency emphysema and smoking cases (55 ± 27 packs-year). For the sake of brevity, the abbreviation AAT-deficiency emphysema for smoking patients with AAT-deficiency will be used throughout the manuscript.

All patients had quit smoking at least 1 year before undergoing surgery.

The average control patient age was 34 ± 16.8 years and cerebral trauma was the cause of death. All the donors stayed less than two days in intensive care without evidence of lung infection or other complications. During artificial ventilation, airway pressure (P_aw_) was 20,9 ± 1.5 mmHg and inspiratory oxygen fraction (FI, O_2_) was 0.4 ± 0.1.

All the samples showed various degrees of emphysematous changes. In particular, all the patients with AAT-deficiency showed diffuse destruction of alveolar tissue, consistent with panlobular emphysema. In contrast, relatively preserved lower portions of the lungs were observed in patients with smoking-associated emphysema, consistent with centrolobular emphysema.

### Immunophenotype analysis

Emphysema patients had an increased number of ICI (CD20, CD3, CD8, CD68, CD45RO, CD4 and PMN) as compared with controls (p ≤ 0.01). An increased number of CD3 (p ≤ 0.05), CD8 (p ≤ 0.05) and CD45RO (p ≤ 0.001) was seen in AAT-deficiency emphysema compared to smoking-related emphysema (Table 2).

**Table 2:** Inflammatory Cells (Total Cells/MM Alveolar Wall)

	**AAT**	**Smokers**	Controls *	***P***†
**CD20**	4.77	2.1	0.0	*ns*
**CD3**	25.39	17.84	2.5	*<0,05*
**CD8**	12.32	5.07	0.99	*<0,05*
**CD68**	4.35	6.88	0.75	*ns*
**CD45RO**	25.42	8.34	2.31	*0,001*
**CD4**	12.1	12.67	1.89	*ns*
**PMN**	18.07	20.78	0.0	*ns*

Definition of abbreviation. PMN: Polymorphonuclear cells

* The values of control group were all statistically significant compared to both emphysema groups.

† Significant differences between AAT-deficiency and smoking patients.

### Analysis of epithelial apoptosis and proliferation

Labeling of the DNA breaks by TUNEL demonstrated positive cells that were localized to peribronchiolar, intra-alveolar and septal sites in both normal and emphysematous lungs. Quantification was limited to the alveolar wall. Apoptotic bodies that were very close to each other were counted as one dying cell. Intra-alveolar apoptotic cells were not included in the cell count.

**Figure 1:**
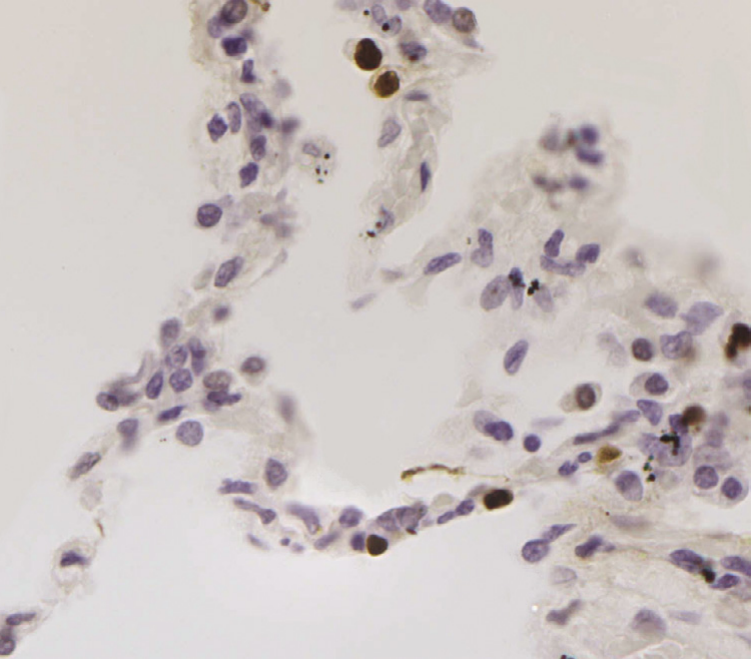
**AAT-deficiency emphysema case 1: **Micrograph showing strong specific staining for DNA strand breaks in the alveolar epithelial cells and in two cells detaching from the wall. TUNEL (original magnification 160×).

**Figure 2:**
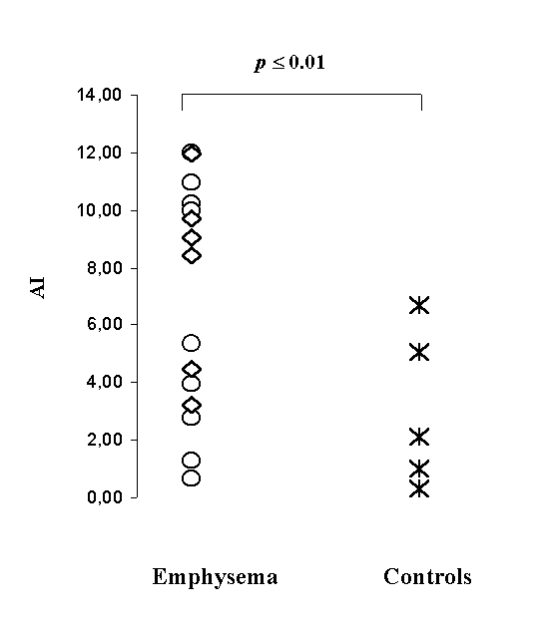
**AI in controls vs emphysema patients: **Significantly higher AI was found in emphysema patients (6.5 ± 3.5 vs 2.7 ± 2.6, p ≤ 0.01) ◊ = AAT-deficiency emphysema; ○ = smoking-related emphysema.

**Figure 3:**
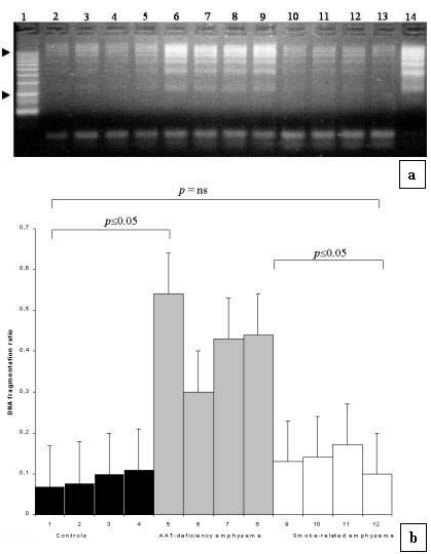
**Gel-electrophoresis: a) **Oligonucleosomal-length DNA laddering in emphysematous and control lungs. Lane 1: DNA marker; Lanes 2–5: control donor lungs (4 cases); Lanes 6–9: AAT-deficiency emphysema patients (4 cases); Lanes 10–13: smoking-related emphysema patients (4 cases); Lane 14: positive control. **b) **Quantification of DNA laddering based on scanning densitometry of bands approximately between 1000 bp and 300 bp (arrowhead) followed by normalization with the density obtained with the equivalent band of the thymus DNA positive control (lung sample/control = densitometric ratio) which was included in every oligonucleosomal DNA laddering assay.

In emphysematous lungs AI ranged from 0.68 to 11.92 (mean 6.3 ± 3.5). The TUNEL-positive cells were more frequently detected within more enlarged alveolar walls. Apoptotic cells and/or bodies were frequently seen in intra-alveolar lumen that presumably represented apoptotic cells detached from the alveolar wall (Fig. 1). AI was significantly higher in patients than in controls (6.5 ± 3.5 vs 2.7 ± 2.6, p ≤ 0.01) (Fig 2). If separately compared with the control group only the AAT-deficiency emphysema showed a statistically significant difference (p ≤ 0.01 vs p = 0.09). Increased levels of oligonucleosomal-length DNA fragments were also detected in emphysema patients, particularly in AAT-deficiency emphysema, than control lungs (Fig. 3a,b). The PI of patients ranged from 0.19% to 4.81% (mean 1.9 ± 2.2). Similar numbers of MIB1-positive alveolar septal cells were observed in both types of emphysema and control lungs (1.7 ± 1.1).

**Figure 4:**
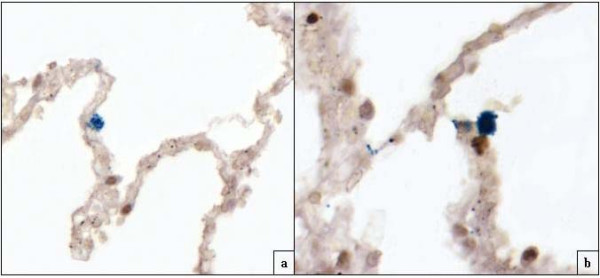
**Smoking-related emphysema case 3: a) **double labeling TUNEL/MIB1 (marker of cell proliferation) showing two apoptotic cells (dark) and one MIB1-positive cell (blue), on the surface of the same alveolar wall (original magnification 160×). **b) **Note alveolar cell in proliferation close (blue) to apoptotic pneumocyte (dark) (Original magnification 160×).

TUNEL-positive/MIB1-negative nuclei detected by double staining were seen in all cases, whereas MIB1 was never expressed in any of the TUNEL-positive nuclei (Fig. 4a,b).

In each group, no statistically significant correlations were found between AI and PI as well as between AI/PI and ICI.

At electron microscopy typical features of early apoptosis with margination of chromatin at the nuclear membrane and late apoptosis with completely dense nuclear chromatin, including apoptotic bodies in various stages of degradation, were seen in pneumocytes, endothelial cells and fibroblasts. Typical features of reduplication of vessel basal membranes were frequently seen in cases with more evident apoptosis (5 a-f). Ultrastructural analysis showed more frequent mitotic features in type II pneumocytes.

**Figure 5:**
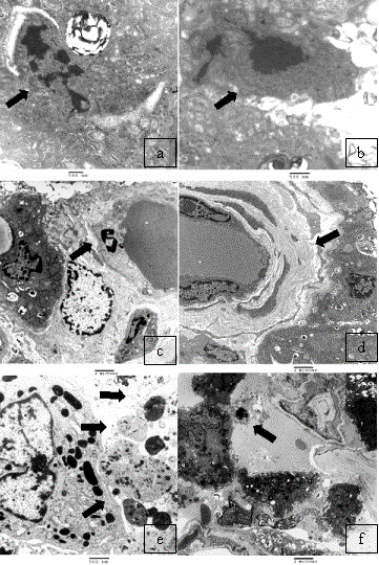
**AAT-deficiency emphysema (case 1): Electron micrograph showing (a) early apoptosis **with perinuclear chromatin condensation (arrow) and (**b) late apoptosis with **nuclear dense chromatin of pneumocytes (arrow). **(c) **An endothelial cell with condensed chromatin is well visible (arrow). **(d) **Note reduplication of the vessel basal membrane (arrow). In **(e) and (f) **apoptotic bodies in various degrees of degradation close to a macrophage and an intraluminal apoptotic body are well visible (arrows).

### TGF-β1 and TGF-βRII receptor analysis

**Figure 6:**
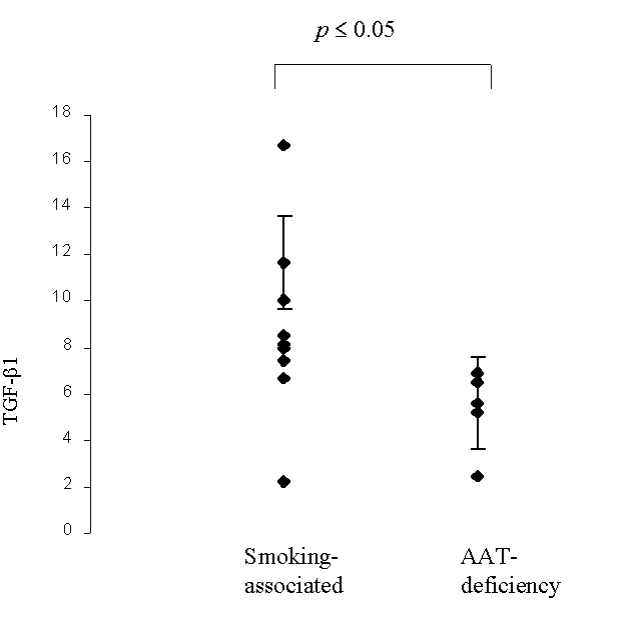
**TGF-β1 expression in smoking-related vs AAT-deficiency emphysema: **the graphic shows the different cytokine expression in both types of emphysema. A significantly higher TGF-β1 expression was found in smoking-related emphysema versus AAT-deficiency emphysema (mean value 8.8 ± 1.7 vs 5.2 ± 3.9, p ≤ 0.05).

**Figure 7:**
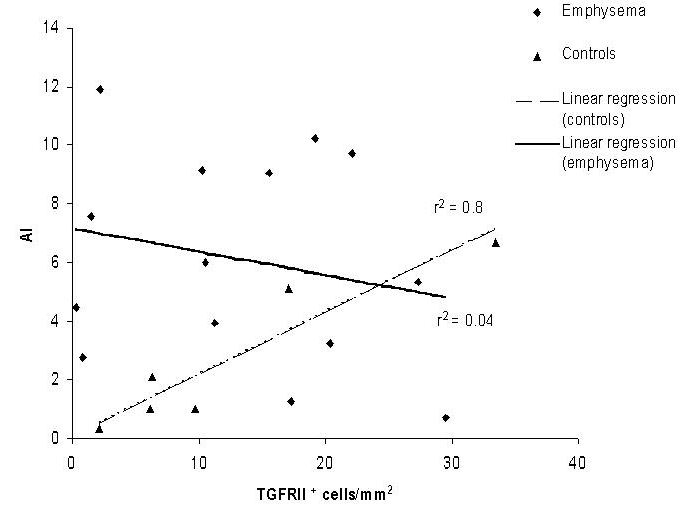
**Correlation between TGF-βRII and AI: **the graphic shows the correlation between TGF-βRII expression and AI in controls and emphysema patients. The degree of TGF-βRII is linearly related to the extension of apoptosis in the control group (p ≤ 0.005, r^2 ^= 0.8).

In emphysema patients and controls TGF-β1 and TGF-βRII were localized in bronchiolar and alveolar epithelial cells and macrophages. Quantitative analysis of TGF-β1 measured in the alveolar wall showed no statistically significant difference between emphysema patients and controls. A higher cytokine expression was noted in patients with smoking-associated emphysema compared with AAT-deficiency disease (mean 8.8 ± 1.7 vs 5.2 ± 3.9, p ≤ 0.05) (Fig. 6). A positive significant correlation between TGF-βRII and AI (p = 0.005; r^2 ^= 0.8) was seen in control lungs (Fig. 7). A significant negative correlation was found between TGF-β pathway (particularly TGF-βRII) and T lymphocytes infiltrate (CD3+) (p ≤ 0.05, r^2 ^= 0.99) in smoking-related cases. No correlation was noted between the TGF-β1 pathway (TGF-β1 and its RII) and the AI/ PI of emphysematous lungs.

## Discussion

In the present study we have analyzed for the first time apoptosis and proliferation in different types of end-stage emphysema. The detection of a high AI in emphysematous lungs even in end-stage disease emphasizes the importance of the phenomenon in the development, and overall, in the progression of emphysema.

In general there are two main forms of cell death: oncosis and apoptosis. The latter process results in characteristic biochemical features and cellular morphology such as cell shrinkage condensation and fragmentation of the nucleus into well contained fragments called apoptotic bodies.

Perturbation of normal rates of apoptosis has been implicated in many pathologic conditions such as neuro-vegetative, cardiovascular and liver disorders and cancer [20-22]. As stated in Tuder's recent review on apoptosis and its role in emphysema, cell damage, apoptosis, apoptotic cell removal, and cellular replacement are ongoing and presumably highly regulated in order to maintain homeostasis of the entire alveolar unit. The concept of the irreversible destruction of alveolar walls due to the loss of homeostasis of the alveolar unit is a critical point. Lung inflammation, protease/antiprotease imbalance, oxidative stress and apoptosis could work together in the irreversible changes seen in emphysema [23]. Over-induction of apoptosis and inefficient cellular replenishment, modifying alveolar homeostasis, would both be expected to disrupt the alveolar wall thus inducing the development of emphysema.

Recently the causal role of apoptosis has been increasingly recognized in the destruction of alveolar walls and airspace enlargement [6-9]. Among constitutive cell populations of the alveolar wall, epithelial cells are more frequently susceptible to programmed cell death [6,9]. In our study the AI of epithelial cells was significantly higher in end-stage emphysema cases compared to the control group (p ≤ 0.01) and this was particularly more evident for those with AAT-deficiency.

To avoid a bias of under or over-estimated alveolar cell apoptosis and proliferation due to regional disease activity we analyzed large specimens taken from different lung regions (upper and lower lobes). The lower AI detected in our control lungs underlines an important concept: in non-emphysematous lungs apoptosis is an irrelevant process adequately balanced by proliferation. The increased number of apoptotic cells in patients with emphysema (not adequately replaced by new epithelial cells) suggests a new mechanism, namely "epithelial apoptosis/proliferation imbalance" in the pathogenesis of disease. In our study, different from a recent clinical study by Yokohori et al [9], a PI similar to that of the control group was detected in the alveolar epithelial cells of emphysema patients. The discrepancies between the two studies can be attributed to several factors: 1) a different monoclonal antibody used for detection of cell proliferation (MIB1 vs PCNA); 2) different case series including patients affected by emphysema in end-stage status and overall of different types (smoking-associated and AAT-deficiency emphysema), and 3) more analysis of extensive areas (upper and lower lobes) of emphysematous lung parenchyma. Regarding the monoclonal antibody used for proliferation detection, Ki-67 is now well recognized as the most reliable immunohistochemical marker for the analysis of cell proliferation in formalin-fixed, paraffin-embedded tissue [24]. Immunoassaying for proliferating nuclear cell antigen (PCNA) can also be used in paraffin-embedded tissue, but it may overestimate the proliferation rate given the long half-life of this antigen [25]. Moreover, the simultaneous positive staining of TUNEL and PCNA in the same cells has been reported [26]. In fact, it has also been demonstrated that PCNA expression can increase without a corresponding increase in S-phase DNA synthesis [27].

DNA nicks may be seen in cells with DNA synthesis/repair thus sometimes producing false TUNEL positive cells. False positive TUNEL staining can also be generated through non-apoptotic mechanisms: RNA synthesis and splicing, necrosis as well as artifacts due to preservation methods. Consequently, some authors have stressed the importance of associating other techniques, such as *Taq *polymerase-based DNA *in situ *ligation, DNA gel electrophoresis or electron microscopy, in order to avoid false positive labeling and to assess the reliability of apoptosis [28].

Our TUNEL findings have been corroborated by employing an additional quantitative apoptosis assay. Moreover, the presence of different stages of apoptosis was confirmed and the cells involved in programed cell death were well characterized by using electron microscopy investigation, which is considered the gold-standard technique for apoptotic cell detection. In our work double-immune labeling showed that all TUNEL positive cells were consistently negative for MIB1 thus suggesting the true epithelial DNA fragmentation. Although the high AI detected in our patients could be mainly explained by the high rate of apoptotic cell death, an impaired clearance mechanism of apoptotic cells/bodies should also be considered. A frequent finding of apoptotic bodies in alveolar walls and within lumen may support the latter theory as an important contributing factor for a high percentage of AI.

The principal trigger of epithelial injury leading to apoptotic cell death is up to now still unclear. The cytotoxic effects of cigarette smoke, one of the most clearly proven etiologic factors in the development of emphysema and in general of COPD, have been suggested to suppress epithelial proliferation and to induce cell death. In particular oxidants and aldehydes, major constituents in the volatile phase of cigarette smoke, have been reported to induce apoptosis of lung cells [29].

Different DNA and RNA viruses have been identified as viral pathogens associated with the disease. Double-strand DNA viruses such as adenovirus have the ability to persist in airway epithelial cells long after the acute infection has cleared. The expression of adenoviral trans-activating proteins has been demonstrated in the airway epithelium of both human and animal lungs and is associated with an amplification of cigarette smoke-induced inflammatory response [30].

Different adenovirus early proteins, in particular E4orf4, have been reported in the shutoff of host protein synthesis and in the promotion of a p53-independent cell death program [31]. It is likely that many and various noxious agents all come together to play an important role in the progression of cell death in end-stage disease, justifying the high AI in the alveolar wall, as detected in our study. The specific molecular pathogenetic pathways that regulate both cell fate and proliferation are also under investigation. Previous studies demonstrated an inhibitory effect of the TGF-β1 pathway on the growth of lung epithelial cells [14,15].

As the TGF-β1 pathway is well-known for its anti-inflammatory activity, a higher epithelial expression of TGF-β1 in patients with smoking-related emphysema compared with AAT-deficiency may partially justify the different patterns of inflammation in the two types of emphysema, as found in our study. A significantly higher increase of inflammation, particularly due to T lymphocytes, was found in AAT-deficiency emphysema (panlobular type) than in smoking-related disease (centrilobular type), with the latter displaying an increased expression of the TGF-β pathway (as demonstrated by the negative correlation with T lymphocyte infiltrate). Similar findings have been previously reported in both *in vitro *and *in vivo *studies [32,33]. An increased pro-apoptotic milieu of inflammatory related cytokines may contribute to the higher cell death rate detected in AATdeficiency emphysema. Moreover, additional cigarette smoke-mediated damage should also be considered in AAT-deficiency emphysema patients, in that in our study they were all heavy smokers.

In our work a direct correlation between TGF-βRII and AI was found in the control group thus showing that this cytokine could play a role in alveolar homeostasis in physiologic conditions. Instead no correlation was found between the AI and TGF-β1 pathway in either type of emphysema, suggesting that the TGF-β1 regulated mechanism is lost in the disease. Other cytokines besides TGF-β1 could be involved in uncontrolled programed cell death inducing the progressive disappearance of the alveolar unit.

Decreased expression of VEGF and VEGF R2 has been demonstrated to be significantly correlated with apoptosis of both epithelial and endothelial cells in cigarette smoking-induced emphysema [7]. It has been shown that VEGF receptor signaling is extremely important for the maintenance of alveolar structures. Hence an impairment of its trophic endothelial activity may be one of several factors facilitating alveolar septal cell apoptosis [4,7]. Significantly reduced levels of VEGF have also been detected in induced sputum of emphysema patients compared to that of normal individuals and patients with asthma [34].

More recently in an experimental model some authors have shown that over-expression of placenta growth factor (PIGF) causes a phenotype and pulmonary dysfunction similar to human lung emphysema by inducing apoptotic events in the alveolar septa [35]. Although epithelial cells have been demonstrated to be more susceptible to apoptosis [7], endothelial cells are also an important target for programed cell death. Our ultrastructural analysis showed evidence of endothelial cell apoptosis mainly in those cases with more increased alveolar programed cell death. The presence of a multi-layered vessel basement membrane, as found in many of our emphysematous lungs, may also reflect additional data supporting the increased apoptotic rate of endothelial cells.

In summary, our work has demonstrated for the first time that apoptotic phenomenon is extensive also in end-stage emphysema patients. This unique case series, and overall the large variety of lung tissue samples examined, (not only subpleural emphysematous regions as those from lung volume reduction surgery in which apoptosis could already be switched off) may account for the differences in our AI findings compared to other studies [9]. The higher rate of apoptotic cell death in patients with AAT-deficiency emphysema, partially influenced by the higher degree of inflammation, may allow us to consider this peculiar emphysema subtype as an additional modifier of apoptosis.

Whether the "apoptosis/proliferation imbalance" occurs before, after or at the same time as the "elastase/antielastase imbalance" is still unknown and should be the subject of future studies.

### Limitations of the study

Our study had a few limitations. Firstly, the patients with AAT-deficiency can not be considered pure AAT-deficiency emphysema cases because these patients were also smokers. Panlobular emphysema occurs at a younger age in alpha-1-antitrypsin patients, especially if the patients smoke cigarettes, as in our case series (49.8 ± 5.7 yrs AAT-deficiency vs 58.2 ± 6.3 yrs smokers, p ≤ 0.01). Patients with AAT-deficiency who are smokers develop lung impairment function earlier and in a more severe form than their non-smoking counterparts. Thus, it is extremely rare to have patients who are non-smokers with AAT-deficiency as candidates for lung transplantation. Secondly, the clinical characterization of the donor was poor and according to the guidelines for the selection of donor lungs, smokers were not excluded [36]. Smokers could have been included in the control group, and it is well known that smoking itself may induce apoptosis.

However, if this was the case, the AI difference between emphysema and control patients would have been even higher because of the lower AI in healthy patients. A third potential bias is that all the donors were mechanically ventilated before lung transplantation and it is known that mechanical ventilation may induce lung apoptosis [37]. Again, the difference observed in our study would be even higher than non-ventilated controls, thus confirming the findings that enhanced apoptosis may act as a leading mechanism in the pathogenesis of emphysema.

## Conclusions

Our study analyzed apoptosis and proliferation in end-stage emphysema. In particular the work described for the first time a high AI in patients with AAT-deficiency emphysema. Ultrastructural investigation, TUNEL analysis and oligonucleosomal-length DNA laddering, performed in different lung regions were all used for detection of apoptotic phenomenon. The increase of apoptotic cells in patients with emphysema not adequately replaced by new epithelial cells suggests a new mechanism, namely "epithelial apoptosis/proliferation imbalance" in the pathogenesis of disease. More inflammation, particularly due to T lymphocytes, was observed in AAT-deficiency emphysematous lungs. An increased pro-apoptotic milieu of inflammatory related cytokines may contribute to the higher cell death rate detected in AAT-deficiency emphysema. While a direct correlation between TGF-βRII and AI was found in the control group, no relation was found between the AI and TGF-β1 pathway in end-stage emphysema, suggesting that the influence of the TGF-β pathway on epithelial turnover is lost in the disease. Knowledge of the mechanism responsible for activation and progression of the apoptotic cascade could offer new information in the near future, on more appropriate stratification and treatment of the disease.

## Authors' contributions

FC: conceived of the study and participated in its design and coordination

CG: substantial contribution in study design and data interpretation

BB: acquisition of clinical data and critical revision for important intellectual content

FR: thoracic surgeon providing lung specimen and critical revision for important technical aspects

ML: thoracic surgeon providing lung specimen and critical revision for important technical aspects

RZ: critical revision for important intellectual content

GP: acquisition of clinical data

SB: acquisition of clinical data and performed the statistical analysis

MS: participated in the design of the study and gave critical revision for important intellectual content

MV: substantial contribution in study design and data interpretation

All the authors read and approved the final manuscript.
